# Peptide‐based supramolecular hydrogels for delivery of biologics

**DOI:** 10.1002/btm2.10041

**Published:** 2016-11-09

**Authors:** Yi Li, Feihu Wang, Honggang Cui

**Affiliations:** ^1^ Dept. of Chemical and Biomolecular Engineering The Johns Hopkins University 3400 N Charles Street Baltimore MD 21218; ^2^ Institute for NanoBioTechnology, The Johns Hopkins University 3400 N Charles Street Baltimore MD 21218; ^3^ Dept. of Oncology and Sidney Kimmel Comprehensive Cancer Center The Johns Hopkins University School of Medicine Baltimore MD 21205; ^4^ Center for Nanomedicine The Wilmer Eye Institute, The Johns Hopkins University School of Medicine 400 North Broadway Baltimore MD 21231

**Keywords:** biologics, gene therapy, peptide, protein delivery, supramolecular hydrogels

## Abstract

The demand for therapeutic biologics has rapidly grown over recent decades, creating a dramatic shift in the pharmaceutical industry from small molecule drugs to biological macromolecular therapeutics. As a result of their large size and innate instability, the systemic, topical, and local delivery of biologic drugs remains a highly challenging task. Although there exist many types of delivery vehicles, peptides and peptide conjugates have received continuously increasing interest as molecular blocks to create a great diversity of supramolecular nanostructures and hydrogels for the effective delivery of biologics, due to their inherent biocompatibility, tunable biodegradability, and responsiveness to various biological stimuli. In this context, we discuss the design principles of supramolecular hydrogels using small molecule peptides and peptide conjugates as molecular building units, and review the recent effort in using these materials for protein delivery and gene delivery.

## 1 Introduction

Biologics, including monoclonal antibodies (mAbs), cytokines, growth factors, enzymes, vaccines, and genes, are biomedical products that are produced or extracted from a living system such as human, animal, or microorganism. In most cases, biologics refer to the biopharmaceuticals produced through recombinant DNA technology. The development and maturation of recombinant DNA technology during the past four decades has led to industrial production of biologics in a cost‐effective manner. In 1982, the first biopharmaceutical Humulin (human insulin) was approved by the U.S. Food and Drug Administration (FDA). Between 1982 and 2012, more than 300 new biological products have received FDA approval for clinical use.^1^ The global biopharmaceutical market has claimed an increasing share of the total global pharmaceutical market, comprising 20% of the market in 2014 and projected to reach 27% by 2018.^2^ It is now clear that the rapid growth of the biopharmaceutical market is tipping the balance toward heavy investment in therapeutic biologics from both industry and government. The promising therapeutic effects, combined with much reduced toxicity, make them ideal alternatives to traditional medicines for battling many types of diseases. Currently, more than 900 biologics are under development aiming to improve the treatments for a variety of diseases including cancer, infectious diseases, autoimmune diseases, cardiovascular diseases, and so on.^3^

Therapeutic proteins, mainly consisting of mAbs, Fc fusion proteins, recombinant enzymes, and antibody fragments, are the most rapidly growing field in biopharmaceutical industry.^4^, ^5^, ^6^ In general, therapeutic proteins possess superior properties over small molecule drugs, such as highly specific and diverse functions associated with their structural complexity and delicacy that cannot be easily mimicked by synthetic chemistry.^7^, ^8^ However, their large size and complicated structures often cause issues for delivery such as limited solubility, chemical and physical instability, rapid renal clearance, low membrane permeability, and poor tissue penetration.^5^, ^8^ In addition, for systemically delivered therapeutic biologics, an in‐depth understanding of both pharmacokinetics (PK) and pharmacodynamics (PD) is much needed.^9^ Due to the high sensitivity of proteins to the gastrointestinal tract environment, the most preferred route for proteins—oral delivery—is such a complex and challenging issue that no orally taken protein drugs are currently approved by the FDA. In light of this, most protein drugs are administered intravenously, intramuscularly, or subcutaneously. To address the issues in protein delivery, controlled chemical modifications such as substitutions, acylation, and PEGylation have been widely explored to optimize the PK properties. One concern associated with this strategy is the possibilities of altering the bioactivity and potency of the conjugated proteins when auxiliary groups or segments are added. Another methodology that has been extensively investigated is encapsulating therapeutic proteins in well‐defined nanostructures or nanostructured materials such as nanoemulsions, liposomes, polymeric micelles, polymersomes, and hydrogels.^8^, ^10^

Gene therapy is another promising biological therapy that has drawn considerate attention which focuses on diseases that include cystic fibrosis, hemophilia, cancer, AIDS, cardiovascular pathologies and others.^11^, ^12^ As of 2012, more than 1,800 clinical trials on gene therapies had been carried out and the future trial activity is predictably positive.^13^ By directly delivering nucleic acids of selected sequences into cells, multiple therapeutic effects could be achieved including correcting genetic defects, replacing or inactivating mutated genes, or overexpressing desired proteins. Apart from DNA, nucleases and several types of ribonucleic acid (RNA) such as small interfering RNA (siRNA), short hairpin RNA (shRNA) and microRNA (miRNA), may also be used to modulate gene expression.^12^, ^14^ Challenges also remain in delivering the large, fragile, and negatively charged DNA and RNA molecules with short lifetimes.^15^, ^16^ Viral vectors were the first and most widely used vehicles to deliver therapeutic genes with high efficiency, but concerns arose due to unwanted issues with pathogenicity, immune response and inflammatory reactions.^14^ Therefore, non‐viral vectors (e.g., gene gun, liposomes and particle‐mediated gene transfer) have also been developed as safer alternatives.^17^

The continued growth of the biopharmaceutical market necessitates the development of efficient delivery systems that can address the numerous challenges in delivering biologics. For both protein carriers and gene vectors, their introduced delivery systems play a vital role in their PK and drug release profiles, and thus should be at least non‐toxic and biocompatible.^18^ In most cases of employing a nanocarrier, the PK of the entire system is usually determined by the properties of the drug carrier, rather than the drug itself. Carriers loaded with a considerable amount of therapeutic agents are expected to circulate for a sufficient period of time before preferentially accumulating in targeted sites.^19^ For example, polyethylene glycol coated (PEGylated) liposomal carriers can prolong plasma half‐life and prevent rapid renal clearance.^20^ The loaded drugs are expected to release from the carriers with a desired releasing profile that is controlled by the design of the material properties of the carrier. In the cases of local delivery using hydrogels, the drug release from a hydrogel scaffold can be well‐controlled by the mesh size,^5^ the interactions between the drug and the scaffolding material, as well as the degradation rates of the hydrogel. Stimuli‐responsive hydrogels, often referred to as smart hydrogels, have shown great promise due to their high loading capacity, high stability, and responsiveness to biological stimuli such as pH, temperature, ionic strength, enzymes, and redox state.^21^, ^22^, ^23^

**Figure 1:**
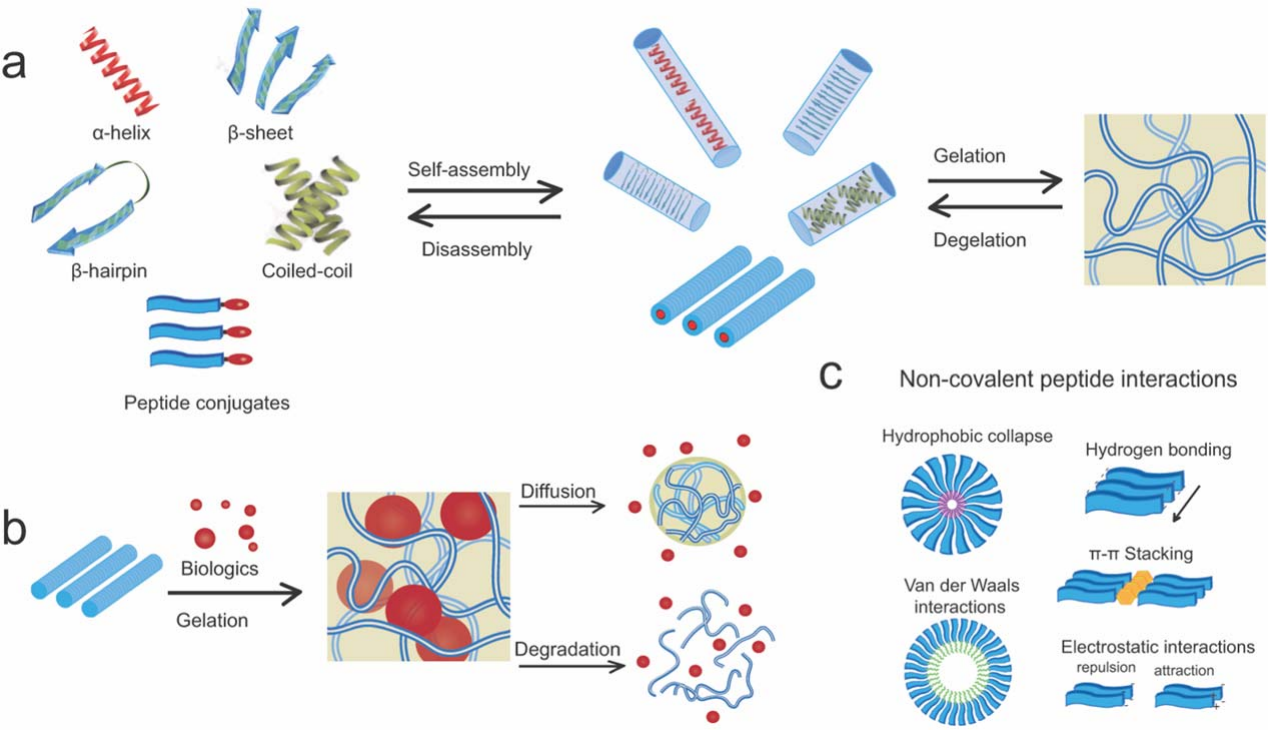
Schematic illustration of (a) the formation of peptide‐based supramolecular hydrogels, (b) the encapsulation and release of biologics using supramolecular hydrogels as carriers, and (c) possible non‐covalent interactions among peptide‐based building blocks, including hydrophobic collapse, Van der Waals interactions, hydrogen bonding, π–π stacking, and electrostatic interactions

The delivery of therapeutics using hydrogels has been extensively explored over the past two decades, mainly focusing on synthetic polymer hydrogels. However, several limiting factors such as component and degradation product toxicity, pains caused by post‐gelation swelling, and short‐term release, remain to be overcome. Among the recently developed hydrogel platforms, self‐assembling peptide nanofibrous hydrogels are particularly fascinating because of their biocompatibility, biodegradability, and low toxicity.^24^, ^25^, ^26^ In general, the peptide‐based supramolecular hydrogels are formed by physical entanglements of filamentous assemblies as a result of several types of non‐covalent interactions among the peptidic building units, which could involve hydrogen bonding, hydrophobic interactions, electrostatic interactions, and π–π interactions (Figure 1a). They display a unique reversibility that most chemically cross‐linked hydrogels do not possess. The entangled networks are able to encapsulate large biologics and offer a stimuli‐triggered and well‐controlled release capability (Figure 1b). Herein, we review the recent progress in the development of peptide‐based supramolecular hydrogels designed for delivery of biologics. The functions and applications of peptide‐based hydrogels in protein drug delivery and gene therapy will be highlighted.

## 2 Design principles of supramolecular nanofiber hydrogels

When dissolved in solvents, amphiphiles such as surfactants, lipids, and amphiphilic block copolymers are able to self‐assemble into various nanostructures (e.g., micelles, nanofibers, nanotubes, vesicles, etc.), with the solvophilic moieties facing towards the solvent and solvophobic moieties packed internally. The final morphology is determined by a balance of the hydrophobic attraction with hydrophilic or ionic repulsion, the system entropy, as well as some directional interactions such as hydrogen bonding or π–π stacking. Among nanostructures of diverse morphologies, one dimensional filaments show great tendency to form supramolecular networks through entanglement or inter filament interactions. Rapid responses to diverse external stimuli are easy to achieve because of the highly reversible property of non‐covalent interactions. Extensive research and reviews have discussed supramolecular hydrogels formed by urea derivatives,^27^ serine‐based amphiphiles,^28^ pyridine‐containing hydrogelators,^29^ multi‐component small molecule systems,^25^, ^27^, ^28^, ^29^ drug amphiphiles,^19^, ^30^ and so on. Peptide‐, nucleobase‐, and saccharide‐based molecules are the three common supramolecular hydrogelators.^31^ The discussion here will focus only on the scope of peptide‐based supramolecular hydrogels.

**Figure 2:**
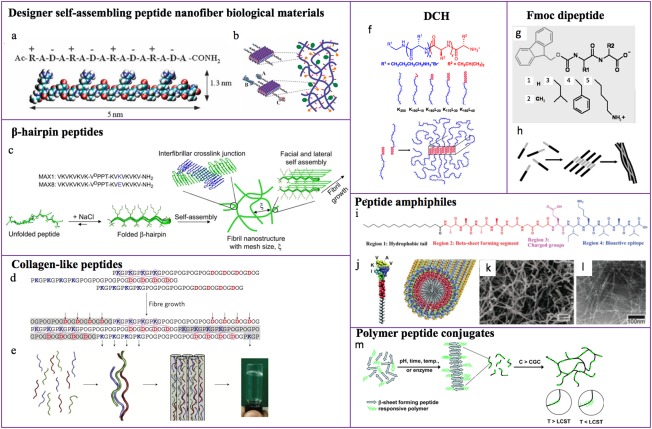
Representative examples of self‐assembling peptide‐based hydrogel systems. (a) Amino acid sequence and molecular model of RADA16‐I. (b) Molecular and schematic models of the RADA16‐I peptides and its assembled scaffolds. (c) Peptide sequences of MAX1 and MAX8, and the proposed mechanism for their self‐assembly into hydrogels. (d) Peptide sequences for collagen‐like peptides. O stands for hydroxyproline. The minimum repeating unit of the triple helical fiber has extensive “sticky” ends. Available interhelical charged‐pair hydrogen bonds are shown by small arrows. (e) Scheme for the self‐assembly of collagen mimetic peptides. The triple helix is staggered with a length of 10 nm and the nanofibers (shown in gray) result from triple helical elongation as well as from lateral packing. (f) Schematic representations of DCH composition, chemcial structures and micellar assemblies. (g) Molecular structure of Fmoc‐dipeptides. The R groups are the amino acids Gly (1), Ala (2), Leu (3), Phe (4), and Lys (5). (h) Proposed self‐assembly mechanism of Fmoc‐dipeptides to form nanofibers. (i) Molecular structure of a representative peptide amphiphile (PA) with four rationally designed chemical entities. (j) Molecular graphics illustration of an IKVAV‐containing PA molecule and its self‐assembly into nanofibers. (k) Scanning electron micrograph of the IKVAV nanofiber network formed by adding cell media (DMEM) to the PA aqueous solution. (l) Transmission electron micrograph of the IKVAV nanofibers. (m) Schematic representation of the self‐assembly mechanism of the *β*‐sheet forming peptide/peptide − polymer conjugate mixtures. (a) and (b) adapted with permission from Ref. 37, (c) adapted with permission from Ref. 32, (d) and (e) adapted with permission from Ref. 51, (f) adapted with permission from Ref. 53, (g) and (h) adapted with permission from Ref. 52, (i)–(l) adapted with permission from Ref. 54, (m) adapted with permission from Ref. 55

With their hierarchically organized structures and the diversity of amino acid function, peptide‐based materials are suitable as building blocks for forming bioresponsive hydrogels. Typical nanofibers formed by self‐assembling peptides have diameters between 5 nm and 15 nm and lengths on the micro‐meter scale, and are able to further entangle to form a 3D network. The inherent biocompatibility and biodegradability are the most fascinating properties for peptidic hydrogels, because the degradation products can be metabolized and reused by cells. Moreover, the well‐established solid‐phase peptide synthesis (SPPS) protocols further contribute to the efficient and easy synthesis of peptides by researchers who have not had rigorous chemistry trainings. The varying functionality of the constituent amino acid side chains within peptides provide a broad basis for non‐covalent interactions including hydrogen bonding (polar amino acids like glutamine), π–π stacking (aromatic amino acids like phenylalanine), hydrophobic collapse (non‐polar amino acids like valine), and electrostatic interactions (acidic and basic amino acids like glutamic acid and lysine) (Figure 1c).^26^ Meanwhile, each type of interaction may respond to different environmental stimuli. Thus one simple sequence‐specific molecular modification may have a huge impact on the physical and chemical properties of the bulk hydrogels.^32^ For example, the solubility of acidic and basic amino acids is determined by the degree of proton dissociation, a property that is pH and ionic strength dependent.^33^ The self‐assembly process of charged peptides can thus be facilitated by tuning the pH or adding salts to reduce the electrostatic repulsions and promote aggregation. Many studies have been carried out on the bioresponsive properties of peptidic systems.^24^, ^26^, ^34^ The design and development of self‐assembling peptide nanofiber hydrogels will be discussed according to the classification of peptides and peptide conjugates. Some representative self‐assembling peptide hydrogel systems are illustrated in Figure 2.

## 3 Peptides

A variety of native peptides are able to self‐assemble into filamentous networks.^25^, ^35^, ^36^ Ulijn and coworkers have recently summarized the bioresponsive elements incorporated in short oligopeptide systems based on different secondary structural motifs including, β‐sheets, β‐hairpins, and helices and coiled‐coils.^26^ For example, the design principles for responsive β‐sheet peptides usually consist of peptide chains with alternating cationic, hydrophobic, and anionic amino acid residues. Inspired by Z‐DNA binding protein zuotin, a number of ionic self‐complementary peptides such as RADA16‐I, RAD16‐II, EAK‐I, and EAK16‐II that can self‐assemble into well‐defined nanofiber scaffolds were designed by Zhang et al.^37^, ^38^ These peptides contain both hydrophilic (charged) side chains and hydrophobic side chains on the different sides of self‐assembled β‐sheet structures.^37^ Under physiological conditions of neutral pH and millimolar salt concentration, millions of β‐sheets pack in parallel and assemble into individual nanofibers that can further form a transparent hydrogel with 99.5–99.9% water content (Figures 2a, b).^37^ Both small molecules and proteins were utilized to show the sustained release through these peptide scaffolds depending on the molecular characteristics (e.g., size and shape) and scaffold properties (e.g., density of nanofibers).^39^, ^40^ These designer self‐assembling peptide nanofiber scaffolds have been widely used in cell culture, reparative and regenerative medicine, and tissue engineering. Recently, two‐layered injectable self‐assembling peptide hydrogels were used as a carrier for therapeutic antibodies.^41^ The self‐assembling Ac‐(RADA)_4_‐CONH_2_ and Ac‐(KLDL)_3_‐CONH_2_ peptide hydrogels were shown to sustained release human antibodies for a period of over 3 months.

Schneider, Pochan, and coworkers developed a series of β‐hairpin peptides that undergo fibril formation and triggered hydrogelation for various biomedical applications.^42^, ^43^, ^44^, ^45^, ^46^ For example, MAX1(VKVKVKVKV^D^PPTKVKVKVKV‐NH_2_) is composed of high β‐sheet propensity valine and charged lysine residues in an alternating manner.^47^ An intermittent tetrapeptide (‐V^D^PPT‐) was designed to adopt a type II′ turn structure that leads to β‐hairpin formation.^48^ The ability of MAX1 to assemble into a β‐hairpin relies on a pH‐promoted intramolecular folding event.^46^ Under the conditions in which the lysine side chains of MAX1 are largely deprotonated, the intramolecular folding occurs with hydrophobic valine residues and hydrophilic lysine residues lined on the opposite face of the hairpin (Figure 2c). The β‐hairpin structure can be stabilized through subsequent self‐assembly of monomeric hairpins into nanofibers as a result of H‐bond formation between distinct hairpins and hydrophobic association of the valine‐rich faces of hairpins.^46^ Importantly, the β‐hairpin structure can be reversibly unfolded by charge repulsion between ionized lysine side chains through lowering of the pH. These (VK)_n_V^D^PPT(VK)_n_‐based peptide hydrogels can be engineered to be transparent, shear recoverable, injectable and pH or ionic strength responsive, all important properties for biomedical applications.^44^, ^45^, ^49^ The mesh size of the hydrogel could be readily controlled by tuning the peptide concentration and the rate of gel formation. In a typical example, by replacing one lysine in MAX1 at position 15 with a glutamic acid, MAX8 (VKVKVKVKV^D^PPTKVEVKVKV‐NH_2_) was shown to self‐assemble and gelate at a much faster rate than MAX1 and form more rigid gels with smaller mesh sizes.^44^ Release studies were carried out using model dextran and protein probes, indicating the well‐controlled release of macromolecules from β‐hairpin peptide hydrogels.^43^ Recently, MAX8 was shown to be able to deliver nerve growth factor and brain‐derived neurotrophic factor (BDNF), as well as active chemotherapeutics (vincristine).^46^, ^50^

Biomimetic collagen‐inspired hydrogels have been widely developed and applied to cell scaffolding applications.^51^, ^56^, ^57^, ^58^, ^59^ Conticello and coworkers used a synthetic peptide system based on a Gly‐Xaa‐Yaa repeat sequence that can self‐assemble into collagen‐like homotrimeric helices driven through electrostatic interactions.^60^ Hartgerink and coworkers utilized the short collagen‐like peptides (Pro‐Lys‐Gly)_4_(Pro‐Hyp‐Gly)_4_(Asp‐Hyp‐Gly)_4_ to mimic the multi‐hierarchical self‐assembly of a collagen fiber from triple helix to nanofiber and hydrogel (Figures 2d, e).^51^, ^58^ The collagen mimetic peptide is able to exhibit triple helical packing and assemble into nanofiber morphologies in a quasi‐hexagonal fashion under a wide range of buffers and ionic strengths, stabilized by salt‐bridged hydrogen bonds between lysine and aspartate on an adjacent lagging peptide offset by three amino acids.^51^ The collagen mimetic hydrogels formed by the triple helix nanofibers were shown to have similar storage modulus to that typically observed for a natural collagen hydrogel and was temperature sensitive due to the unfolding of the triple helix at 40–41°C.^51^ Later on, by comparing two classes of collagen mimetic peptides with the same composition but different domain arrangements, they found that larger sticky‐ended nucleation domains result in rapid fiber formation and eventual precipitation or gelation.^58^ Yu and coworkers have had considerable success in decorating and functionalizing collagen fibers using (Pro‐Hyp‐Gly)‐based collagen mimicking peptides.^61^, ^62^, ^63^ As a result of rational biomaterials design, these collagen mimicking peptides are expected to play an important role in regenerative medicine and drug delivery applications.

Deming and coworkers have developed diblock copolypeptide hydrogels (DCH) containing charged and hydrophobic segments such as poly(l‐lysine)‐*b*‐poly(l‐leucine) and poly(l‐lysine)‐*b*‐poly(l‐valine) (Figure 2f).^53^, ^64^, ^65^, ^66^ In aqueous solutions, if the hydrophobic domains are sufficiently large, they can adopt *α*‐helices, β‐sheets, or random coil structures depending on the peptide sequence and self‐associate to form fibrillar assemblies that further entangle to form 3D networks.^67^ A detailed understanding of DCH structure–property relationships was established to achieve a high level of control over the gel properties.^67^, ^68^ Polypeptides have shown many advantages including temperature stability, structural tunability, rapid healing after stress, and injectability.^53^ Recently, Sofroniew and coworkers demonstrated that a DCH system can provide significantly longer delivery of nerve growth factor maintaining activity for at least 4 weeks, suggesting that DCH have promise as delivery vehicles for therapeutic applications.^69^

## 4 Peptide conjugates

Amino acids or short peptides conjugated to alkyl chains or aromatic groups have been extensively investigated over the recent decades. Both alkyl chains and aromatic groups serve as the hydrophobic segment to promote the aggregation. Bhattacharya et al. reported the gelation of oil using *N*‐alkanoyl‐l‐alanine amphiphiles in an immiscible system.^70^ Recently, they presented a two‐component hydrogelator composed of an *N*‐C_16_H_33_‐chain‐appended L‐alanine amphiphile and a redox‐active viologen‐based partner.^71^ A lamellar‐type of morphology formed by this two‐component system can lead to redox‐active 3D‐fibrous networks. Another phenylglycine‐based amphiphilic gelator was found to exhibit phase selective gelation within 90 s.^72^ With the increased interactions and stability that π–π stacking can provide, aryl motifs such as ferrocenyl, fluorenyl, naphthyl and phenylalanine have become prevalent components of hydrogelators.^25^ Fmoc, a common amino‐protecting group, has been shown to facilitate the formation of self‐assembled hydrogels (Figures 2g, h). Fmoc‐protected diphenylalanine was one of the earliest reported dipeptides that were able to self‐assembled into fibrillar structures and form a rigid hydrogel with less than 1% peptide material in aqueous solution.^73^ Noting the vital function of the Fmoc group in the gelation, Ikeda et al. proposed to replace this Fmoc group with a stimuli‐triggered degradation unit, such as 6‐bromo‐7‐hydroxycoumarin‐4‐yl methoxycarbonyl (Bhcmoc), to achieve an oxidation and pH responsive hydrogel, BPmoc‐FF.^74^

The Xu Lab has made significant contributions to Fmoc protected amino acids such as Fmoc‐Lys and Fmoc‐phenylalanine.^75^, ^76^, ^77^ Similar with Fmoc, the self‐assembly and hydrogelation of naphthyl‐protected amino acids and peptides hydrogelators were also widely explored.^78^, ^79^, ^80^, ^81^ Ulijn and coworkers have investigated a library of Fmoc–dipeptides including Fmoc‐diglycine, Fmoc‐dialanine, Fmoc‐diphenylanaline, and so on.^52^, ^82^, ^83^ Self‐assembled nanofibers can be easily prepared by suspending the Fmoc‐dipeptides in water, with the aromatic groups sitting in the core driven by π–π stacking. It was found that the conditions under which gelation took place varied with the peptide type and some gels were stable under physiological conditions.^52^ Later, a novel model comprising π–π interlocked β‐sheets architecture was constructed, a design based on the anti‐parallel β‐sheets and anti‐parallel π‐stacking shown by spectroscopy in the self‐assembly of Fmoc‐FF.^82^ Recently, by investigating a series of aromatic short peptides, Ulijn and coworkers demonstrated the nanofibrous networks can form at the organic/aqueous interface of a biphasic solvent system. Hand shaking of this mixture leads to formation of micro‐sized emulsion droplets with exceptional stability and potential applications for drug encapsulation and delivery.^84^ Tovar and coworkers developed a series of “peptide‐π‐peptide” triblock molecules that self‐assemble into fairly uniform tape‐like nanostructures by embedding a diverse range of π‐electron units directly within peptide backbones.^85^, ^86^ These assembled peptide networks can further aggregate into entangled fibrillar superstructures through intimate π–π contacts and electronic delocalization.^85^

Xu and coworkers pioneered the design of β‐amino acid and D‐amino acid derivatives for creating supramolecular hydrogels.^87^, ^88^, ^89^, ^90^, ^91^, ^92^ Such β‐amino acid derived hydrogels were shown to be proteolytically resistant with prolonged bioavailability, yet retained their sensitivity to the enzyme (phosphatase) that triggers their formation.^87^, ^88^ Although the unique stereochemistry of D‐amino acids imparts similar stability with β‐amino acids towards most of the endogenous enzymes, its low cellular uptake remains challenging for biomedical applications.^91^ Xu and coworker have also designed a novel class of supramolecular hydrogelators based on conjugates of nucleobases and short peptides.^93^, ^94^ These nucleopeptides such as nucleobase‐FRGD were shown to exhibit long‐term biostability, resisting degradation by proteinase K. In a recent study, the first use of heteronucleopeptides to generate biocompatible and biostable hydrogels was reported.^95^ Two structurally distinct peptides were selected from the interface of a heterodimer of proteins and then conjugated with nucleobases. Supramolecular hydrogels formed by simple mixing of the heteronucleopeptides in water displayed non‐β‐sheet secondary structures, which may preserve the specific functions of α‐helical and random‐coil motifs.

Peptide amphiphiles (PAs) are peptide conjugates containing one or more linear alkyl chains that structurally resemble small molecule surfactants (Figures 2i–l).^54^, ^96^, ^97^, ^98^, ^99^, ^100^, ^101^ Stupp and coworkers have designed a series of PAs that can self‐assemble into nanofibers that enmesh into hydrogels under the physiological condition. The nanofibers are composed of β‐sheets with alkyl chains packing in the core of the fibers and peptide segment exposed to the aqueous environment.^54^, ^96^, ^97^, ^99^ A variety of peptide sequences can be ideally engineered into the peptide region of the PA to realize desired functions such as solubility enhancement or cell adhesion.^54^, ^97^ Self‐assembly of PA molecules into nanofiber matrices could be mediated by metal ions^102^ or aided by charged amino acid residues.^103^ In another work, tubular hydrogels formed by circumferentially aligned peptide amphiphile nanofibers were shown to encapsulate vascular cells and direct cellular organization.^104^ Recently, C_16_‐V_2_A_2_E_2_‐NH_2_ PA hydrogels were used as a sonic hedgehog protein delivery system for the treatment of erectile dysfunction, suggesting the PA hydrogels have potentially broad applications as protein vehicles.^105^ Tirrell and coworkers designed pH‐responsive branched peptide amphiphile composed of histidine and serine amino acids conjugated to a palmitoyl tail.^106^ These PA solutions are able to switch from viscoelastic liquids to an injectable tissue scaffold above pH 6.5 as a result of the protonation of histidine. More recently, a PA hydrogel consisting of C_16_GSH was optimized to have greater utility for peripheral nerve repair compared with a commercially available collagen gel.^107^

The peptides or polypeptides conjugated to a polymer have been shown to be effective molecular gelators. Peptide–polymer conjugates combine the desired functionality of peptides and synthetic polymers.^108^, ^109^ For example, a diblock or triblock peptide‐PEG hydrogel system with peptide segment were designed from the coiled coil region of fibrin.^110^ By studying the FEFEFKFK and poly(N‐isopropylacrylamide) conjugates, Maslovskis et al. found that the fibrillar network and the lower critical solution temperature of the polymer were not affected by each other.^55^ However, the interaction between the polymer and peptide will significantly influence the polymer conformation and the mechanical properties of the hydrogels. Recently, Chmielewski and coworkers designed a thermosensitively tunable hydrogel using collagen‐mimetic peptides and PEG star polymer, suggesting potential applications in protein delivery and tissue regeneration.^111^

Although all of these above‐mentioned peptide‐based hydrogel systems have unique design principles and properties, what remains essentially the same is the spontaneous or triggered hydrogelation after self‐assembly driven by supramolecular interactions, which provides the foundation for delivery of biologics. Of particular note is that these peptide systems themselves can be used as bioactive agents by incorporating or directly using therapeutic or other functional peptide sequences into the hydrogel networks.^112^, ^113^, ^114^, ^115^, ^116^ The incorporation of the well‐known cell adhesion motif RGD into the peptide nanofibers has been investigated by many research groups.^117^, ^118^, ^119^ In one example, self‐assembled nanofibers with bioactive signal displayed on the surface are shown to promote adhesion of encapsulated dermal fibroblasts with subsequent cell spreading and proliferation.^120^ Studies on the significant immunogenicity of the self‐assembling peptide (such as OVA‐Q11) as well as other nonimmunogenic peptide by Collier and coworkers showed broad applications in modulating adaptive immune responses.^121^, ^122^ Other protein‐derived sequences such as IKVAV sequence have also received much attention to functionalize the peptide networks.^123^, ^124^ These peptide‐based active groups may not be classified as “biologics” in terms of their chemical origins, relatively smaller size, simpler structures, and easily controlled chemistry. In fact, peptides bridge the gap between small molecules (typically <500 Da) and biologics (typically >5,000 Da) and can potentially combine the advantages of small molecules and biologics, offering specificity and potency similar to larger biologics but ease for synthesis.^125^ However, the discussion on the small peptide‐based therapeutics is beyond the scope of this review.

## 5 Protein delivery

Challenges in the delivery of therapeutic proteins derive from their large size and complicated structures that lead to limited solubility, stability, and membrane permeability. Desirable properties for the ideal protein delivery vehicles include high protein loading and biocompatibility; the ability to shield the proteins from enzymatic degradation and rapid clearance; the function to provide sustained release in a desired and therapeutically effective way. Among many kinds of delivery systems, the self‐assembling peptide nanofibrous hydrogels are one of the most fascinating candidates for protein delivery applications. The high‐water content in peptide‐based supramolecular hydrogels provides the open space to store biologics. Either hydrophilic or hydrophobic molecules could be entrapped in the inter‐ or intrafiber areas, greatly increasing the solubility of biologics to be delivered. Generally, biologics can be directly encapsulated into the hydrogel during the self‐assembly and gelation process by simply mixing with hydrogelators under mild conditions. The peptide‐based supramolecular hydrogels are supposed to ideally mimic the extracellular matrix and expected to be more biocompatible than polymer hydrogels. This provides the foundation for protection and stabilization of biologics to avoid their inactivation before they reach the desired location. Based on their potential in presenting sustained release, self‐assembling peptide hydrogels are of high interest to be engineered to optimize the release profile of proteins in a desirable fashion. Although, the delivery of small molecule drugs using supramolecular hydrogels have been largely reported, peptidic hydrogel based delivery of macromolecules such as proteins and gene therapeutics is still in its infancy. Recent advances in protein delivery using self‐assembling peptide hydrogels and corresponding applications are summarized in Table 1.

**Table 1:** Recent advances in protein delivery using self‐assembling peptide supramolecular hydrogels

Encapsulated proteins	Peptide category	Peptide sequence	Function or application	Ref.
IGF‐1	Biotinylated RAD16‐II	Biotinylated Ac‐RARA‐DADARARADADA‐biotin	Cell therapy for myocardial infarction	126
Lysozyme, trypsin inhibitor, BSA, and IgG	Ionic self‐complementary peptide	RADA16‐I (Ac‐(RADA)_4_‐CONH_2_)	Sustained release of proteins	40
Human recombinant BDNF, βFGF and VEGF121	Ionic self‐complementary peptide	(a) RADA16‐I (Ac‐RADARADARADARADA‐CONH_2_), (b) RADA16‐DGE (Ac‐RADARADARADARADAGGDGEA‐CONH_2_), and (c) RADA16‐PFS (Ac‐RADARADARADARADAGGPFSSTKT‐CONH_2_)	Regenerative medicine applications	127
Lactoferrin	β‐hairpin	MAX1(VKVKVKVKV^D^PPTKVKVKVKV‐NH_2_) and MAX8 (VKVKVKVKV^D^PPTKVEVKVKV‐NH_2_)	Sustained release of proteins	8
α‐Lactalbumin, myoglobin, and lactoferrin	β‐hairpin	HLT2(VLTKVKTKV^D^P^L^PTKVEVKVLV‐NH_2_) and VEQ3(VEVQVEVEV^D^P^L^PTEVQVEVEV‐NH_2_)	Electrostatic‐based control over protein release in physiological conditions	128
VEGF and FGF‐2	HBPA	C16AAAAGGGLRKKLGKA	Islet transplantation	129
NGF	DCH	K_180_L_20_ and E_180_L_20_	Protein delivery in CNS	69
NGF and BDNF	β‐hairpin	MAX8 (VKVKVKVKV^D^PPTKVEVKVKV‐NH_2_)	Treatments ofspinal cord injuries	49
Human IgG	Ionic self‐complementary peptide	Ac‐(RADA)_4_‐CONH_2_ and Ac‐(KLDL)_3_‐CONH_2_		41
Insulin	Ionic self‐complementary peptide	RADA16‐I (Ac‐(RADA)_4_‐CONH_2_)	Insulin delivery for subcutaneous injection	130
EGFP	Naphthyl‐protected peptide	NapGFFY(X)ssEE, X=K, E, S	Intracellular protein delivery	131, 132
Sonic hedgehog	Peptide amphiphile	C_16_‐V_3_A_3_E_3_‐COOH and C_16_‐V_2_A_2_E_2_‐NH_2_	Prevention of erectile dysfunction	105

The largest and fastest growing protein therapeutics in the United States are antibody‐related drugs for the treatment of many diseases such as cancer, chronic inflammatory disease, cardiovascular, and infectious diseases.^6^ Local and sustained antibody release by hydrogels can reduce both the associated toxicity and the frequent dosages necessitated by the limited life time. The self‐assembling peptide hydrogels are of great promise to serve as the carriers of therapeutic antibodies. However, very few peptide‐based hydrogels have been reported for this application thus far. Lysozyme, trypsin inhibitor, BSA, and IgG were utilized to study the release from RADA16 systems developed in the Zhang Lab.^40^ The release rate was sensitive to the physical size of the proteins, resulting in the slowest release of IgG (more than 60 h before reaching a steady concentration) among the examined proteins due to possessing the largest molecular weight. CD spectra confirmed the nearly identical structures of IgG released from the hydrogel with the native IgG. The monoclonal IgG used here is specific for the 1D4 sequence at the *C*‐terminus of native bovine rhodopsin. A comparison of the association constant *k_a_* or dissociation constant *k_d_*, the binding affinity between the monoclonal IgG and rhodopsin (antigen) did not change significantly before and after being released through the peptide hydrogel for 48 h. This result suggested that the functionality of IgG is maintained during the release from the peptide hydrogel. In a subsequent study, Zhang and coworkers reported a 3‐month study on the release kinetics for human IgG through a two‐layered hydrogel structure with an Ac‐(RADA)_4_‐CONH_2_ core and Ac‐(KLDL)_3_‐CONH_2_ shell.^41^ This two‐layered hydrogel system formed by a two‐step gelation process allows for 100% IgG loading efficiency because of the high‐water content (up to 99.5%). Results showed a sustained release of human IgG over 3 months without compromising its biological activity.

## 6 Growth factors

Growth factors are proteins that are able to stimulate cellular growth and differentiation and regulate a variety of cellular processes, making them useful in tissue engineering and regenerative medicine. For this reason, they are also one of the most commonly investigated protein classes delivered by self‐assembling peptide hydrogels, given the widespread use of hydrogels in this field.

**Figure 3:**
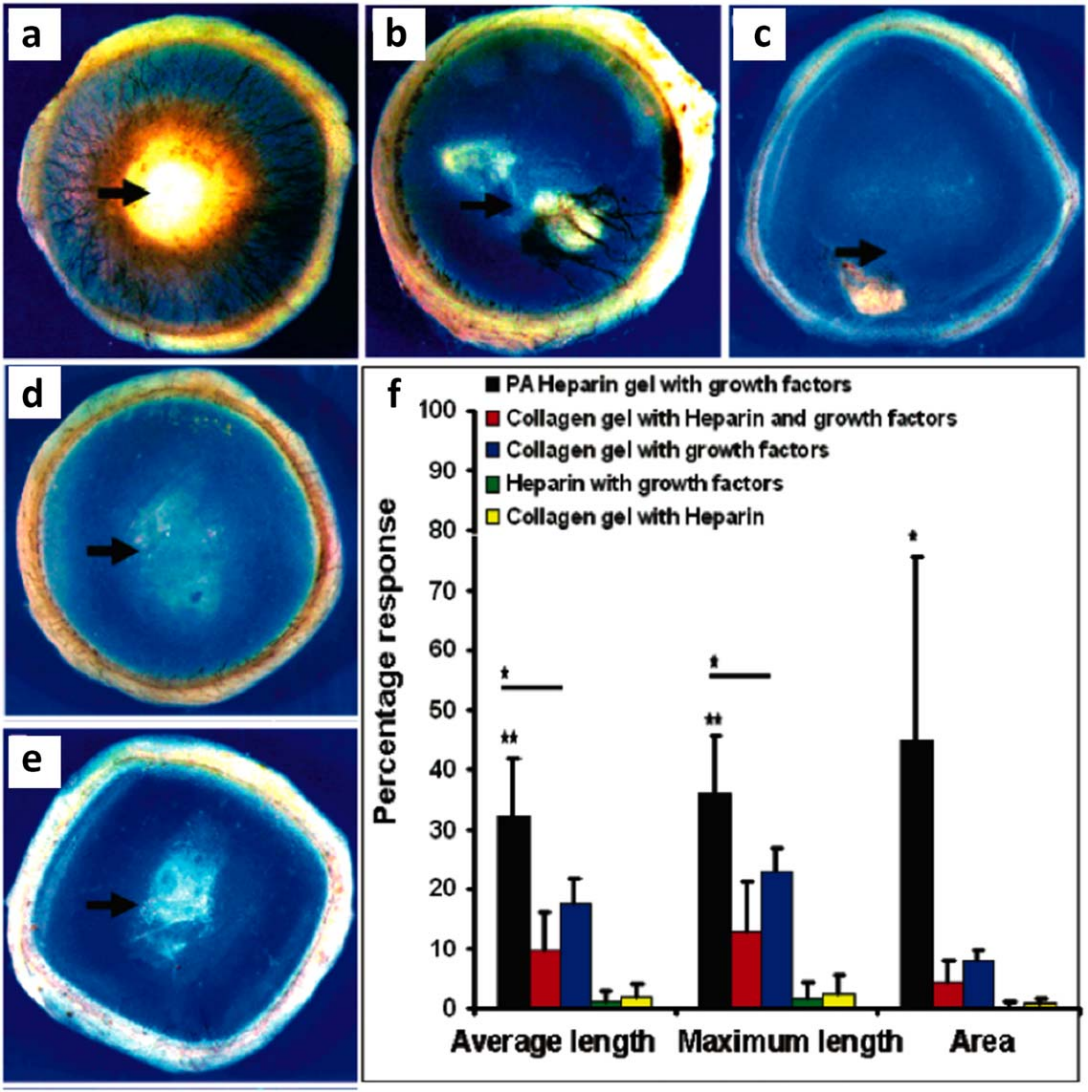
The use of heparin‐binding peptide amphiphile for corneal angiogenesis in a rat model. Photographs of a rat cornea 10 days after the placement of the HBPA system at the site indicated by the black arrow. (a) Heparin‐nucleated PA nanofiber networks with growth factors show extensive neovascularization. Controls of collagen, heparin, and growth factors (b) and collagen with growth factors (c) show some neovascularization. Heparin with growth factors (d) and collagen with heparin (e) showed little to no neovascularization. The bar graph (f) contains values for the average and maximum length of new blood vessels and the area of corneal neovascularization. PA nanofibers with heparin, PA solution with growth factors, and growth factors alone did not result in measurable neovascularization. Used with permission from Ref. 114

The peptide amphiphile (PA) system developed in the Stupp lab serves as a natural and powerful platform for delivery of growth factors and related cells. 54, 102, 105, 112, 113, 129, 133, 134, 135, 136, 137 In one example, heparin‐binding peptide amphiphile (HBPA) was designed with a heparin‐binding domain to specifically bind heparan sulfate‐like gylcosaminoglycans (HSGAG).^114^ This particular design provides the resultant supramolecular nanofiber the capability to recruit proteins possessing the heparin‐binding domains such as fibroblast growth factor 2 (FGF‐2), bone morphogenetic protein 2 (BMP‐2), and vascular endothelial growth factor (VEGF). As a result of charge neutralization of the positively charged HBPA by the negatively charged heparin, the binding event initiate the self‐assembly of HBPA into a nanofiber hydrogel. Given the important role of FGF‐2 and VEGF in angiogenesis, the HBPA has been explored to induce corneal angiogenesis in a rat model (Figure 3). This HBPA system was also used for islet transplantation through the co‐delivery of VEGF and FGF‐2.^136^ The scaffolds were prepared in fibrous poly (l‐lactic acid) (PLLA) matrices by adding a certain amount of aqueous HBPA, and a heparin/growth factor mixed solution, and were transplanted adjacent to islets. The experimental results showed that the HBPA scaffolds containing VEGF/FGF‐2 could significantly increase neovessel densities in the mouse omentum compared with control scaffolds without growth factors. Meanwhile, the cure percentage of the diabetic mice that treated with HBPA scaffolds containing VEGF/FGF‐2 (78%) was more than twice that of control animals treated with scaffolds alone (30%) or growth factors alone (20%). This indicated the effective delivery of growth factors through the HBPA scaffolds contributed to the enhanced engraftment. In a subsequent study, the infiltration of the same HBPA nanofibers into the dense islet interior was achieved by using a 100 times diluted HBPA solution, suggesting the amplified signaling of angiogenic factors leads to an increase in both islet survival and insulin secretion.^129^

**Figure 4:**
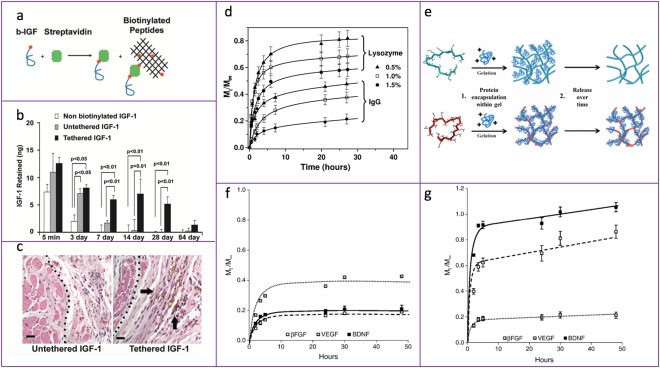
(a) Schematic illustration showing the biotin sandwich method of tethering biotinylated IGF‐1 (b‐IGF‐1) to biotinylated self‐assembling peptides using tetravalent streptavidin. (b) Quantitative delivery of human IGF‐1 to the myocardium of rats. (c) Immunohistochemistry showing phospho‐Akt‐specific staining in untethered b‐IGF‐1 (Left) and tethered b‐IGF‐1 (Right) samples. Arrows denote areas staining positive for phospho‐Akt (brown staining). Scale bars: 20 μm. (d) Release profiles for lysozyme and IgG through Ac‐(RADA)_4_‐CONH_2_ hydrogels of different nanofiber densities with different peptide concentrations. (e) Positively charged protein is encapsulated within an electropositive HLT2 (teal) or electronegative VEQ3 (red) network during gel formation. (f) Cytokine factor release profiles from hydrogel scaffolds composed of RADA16‐DGE (−). βFGF (+) and BDNF (+) were suppressed compared to VEGF (−). (g) Cytokine release profiles from hydrogel scaffolds composed of RADA16‐PFS (+). VEGF (−) was released more slowly than the other protein systems. (a–c) adapted with permission from Ref. 126, Copyright © 2006 National Academy of Sciences, (d) adapted with permission from Ref. 40, (e) adapted with permission from Ref. 128, (f) and (g) adapted with permission from Ref. 127

The Zhang Lab designed the biotinylated RAD16‐II peptide to deliver the cardiomyocyte growth and differentiation factor IGF‐1 to the myocardium to improve cell therapy.^126^ The tetravalent streptavidin was used to bind with both biotinylated RAD16‐II peptides and biotinylated IGF‐1 via a biotin sandwich strategy (Figure 4a), which was found to have no influence on the self‐assembly process of the peptides. The IGF‐1 was observed to be sustainably released for more than 28 days without compromising bioactivity, and increased the cross‐sectional area of cardiomyocytes by 25% compared with cells embedded in hydrogels alone or with untethered IGF‐1 (Figures 4b, c). To achieve better control over the protein release through the peptide nanofiber networks, it is crucial to understand the effect of the physicochemical properties of both proteins and nanofibers on release kinetics. In another study, a variety of proteins including lysozyme, trypsin inhibitor, BSA, and IgG were utilized to determine the relationship between protein release kinetics and protein size through RADA16‐I systems by single‐molecule fluorescence correlation spectroscopy (FCS).^40^ It was demonstrated that protein diffusion would decrease with the increase of nanofiber density or protein size (Figure 4d). To investigate the effect of electrostatic interactions between nanofibers and cytokines upon their mobility through the scaffolds, differently charged cytokines of similar size—human βFGF (+), VEGF (‐) and BDNF (+)—were incorporated into neutral, negative or positive RADA16‐I based scaffolds at a solution pH of 7.4.^127^ Results showed that these cytokines could be slowly and sustainably released from modified RADA16‐I peptides scaffolds and that release of charged proteins would be suppressed when encapsulated within oppositely charged scaffolds compared to similarly charged scaffolds (Figures 4f, g). However, the strategy of using electrostatics to control the release profile of proteins can be hindered by effective charge screening under physiological solution conditions. Nagy‐Smith et al. resolved this issue by designing two highly positive or negative charged peptide hydrogels that were not significantly affected by the physiologically relevant ionic strength, allowing effective electrostatic‐based control over protein release (Figure 4e).^128^

The diffusion of mobile proteins in the hydrogel networks could be hindered both sterically and electrostatically. In the β‐hairpin peptide hydrogel system developed by Schneider and Pochan, a clear relationship between protein release kinetics and hydrogel mesh size as well as electrostatic interactions was demonstrated by studying the release profile of dextran and protein lactoferrin through MAX1 or MAX8 hydrogels.^43^ It was also observed that the obvious influences of any physical interactions on the protein mobility only took place when the protein size approached the mesh size. Subsequently, they investigated the release of model proteins with varying hydrodynamic diameter and charge from MAX8 hydrogels.^138^ From the partition and retention studies, two distinct populations of encapsulated proteins were confirmed to exist in the hydrogel networks: a mobile phase with sterically dictated release and an immobile phase greatly trapped by attractive electrostatic interactions. A similar release profile of the proteins was also found between the gels delivered via syringe and non‐sheared gels.

**Figure 5:**
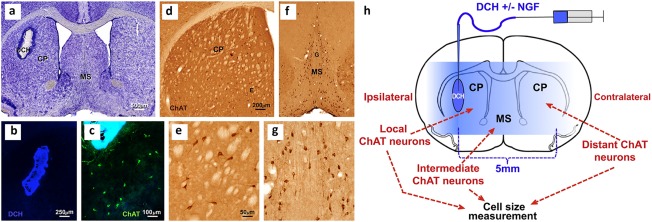
Local delivery of nerve growth factor into the mouse forebrain using the polypeptide assembly system developed in the Deming Lab. (a) A typical DCH depot in the caudate putamen (CP) of mouse forebrain was observed at 1 week after DCH injection. (b) A closer view of DCH depot labeled with conjugated blue fluorescent dye (AMCA‐X) in tissue section. (c) A closer view of the same DCH depot in (b) showing labeled blue DCH plus cholinergic neurons labeled green by immunohistochemical staining for choline acetyltransferase (ChAT). (DeG) Survey (d, f) and detail (e, g) images of ChAT‐stained cholinergic neurons in the CP and medial septum (MS) of an untreated (non‐injected) mouse. (h) Schematic illustration of experimental design to evaluate release of bioactive protein from DCH depots in vivo. NGF was used as a bioactive protein known to induce hypertrophy of basal forebrain cholinergic neurons in the caudate putamen (CP) and medial septum (MS). Depots of DCH with NGF were injected into the CP on one side. Adapted with permission from Ref. 69

Nerve growth factor (NGF) is a neurotrophic factor that has been extensively explored in various applications including tissue engineering and gene therapy. Sofroniew, Deming, and coworkers have investigated the local delivery of lysozyme and nerve growth factor (NGF) for both *in vitro* and *in vivo* tests using the polypeptide assembly system developed in the Deming Lab (Figure 5).^69^ The well‐established structure–property relationships of DCH (either K_180_L_20_ or E_180_L_20_) are able to confer the readily tuned architecture of DCH that can optimize the effect of protein delivery. Results showed that DCH could be safely injected into the central nervous system without unwanted side effects and could deliver bioactive protein inside the blood brain barrier. The DCH scaffolds were proved to provide a temporary gradient of NGF over several millimeters and significantly longer delivery in comparison with injecting NGF solution alone, indicating the high promise of DCH to deliver therapeutic proteins in the CNS and other systems. The release of NGF from self‐assembled β‐hairpin hydrogels has also been reported by Langhans, Pochan, and Schneider.^49^ As is shown from previous studies, the sequence of MAX8 with positively charged lysine side chains allows for triggered hydrogelation.^44^ It could maintain a solution state under the conditions of pH 7.4 with low ionic strength, while raising the pH or salt concentration will lead to the formation of rigid and viscoelastic hydrogels due to screening of electrostatic repulsions. NGF and BDNF could be delivered through MAX8 hydrogels into cell culture media at a low and steady rate. The protein release profiles were monitored over a period of at least 15 days, allowing a more quantifiable data interpretation. The encapsulated NGF within MAX8 was shown to remain bioactive for at least four weeks, much longer than the half‐life of NGF (3.4 d). The release profiles could be readily tuned by the hydrogel concentration and the dosage of encapsulated NGF or BDNF. Additionally, no significant premature loss of encapsulated proteins was observed during the injection, which was attributed to the increased protection by robust domains within the MAX8 hydrogels.

## 7 Other proteins

Human insulin (Humulin®) is the first recombinant human protein therapeutic derived from recombinant DNA technology. Nishimura et al. investigated the in vitro release profile of insulin from hydrogels formed by the Ac‐(RADA)_4_‐CONH_2_ peptide hydrosol (PuraMatrixTM, PM)^130^ and compared the difference of effects evaluated in rats between the PM hydrosol formulations and Humulin R. A greater efficacy of hypoglycemic action of above 0.25% (*w*/*v*) PM hydrosol formulations was found in hydrogel‐released insulin. Additionally, a PM concentration‐dependent increase of the release profile was unexpectedly observed and was later attributed to the increase in osmotic pressure and insulin concentration caused by the increase of PM concentration. The PM hydrosol containing insulin was also subcutaneously injected into rats and was speculated to transform into a gel in response to the increased ionic strength of phosphate or pH of the physiological environment. The difference in release profiles between PM‐delivered insulin and positive control Humulin R, as well as the PM concentration dependent release that resembled the phenomenon in an in vitro study, confirmed this speculation. The systemic circulation of insulin occurring after subcutaneous administration in rats and its pharmacokinetic profile were also investigated. This in vivo sol‐gel transition of PM hydrosol is favorable for systemic delivery of proteins from supramolecular peptide hydrogels, breaking through the restrictions of regular local delivery.

Yang and coworkers have reported that the differently charged peptides of NapGFFY(X)ssEE (X stands for Lys (K), Glu (E), or Ser (S)) could form supramolecular nanofibers upon glutathione (GSH) reduction.^131^ Recently, the negatively charged protein EGFP with inherent fluorescence was used to study the co‐assembly property with the peptides.^131^, ^132^ It was demonstrated that the supramolecular hydrogels could help the protein across cell membranes. Human cervical carcinoma cells (HeLa) were treated with free EGFP or dispersions of different co‐assemblies and confocal laser scanning microscopy (CLSM) was used to monitor the intracellular distribution of the EGFP protein.^132^ Results show that free EGFP was unable to effectively internalize into the cells, while the formation of a co‐assembly could help the EGFP protein penetrate the cell membranes. This observation shows a promising possibility to increase the membrane permeability of proteins using peptide hydrogels.

Recently, Podlasek and coworkers reported the use of self‐assembling peptide amphiphile nanofiber hydrogels for sonic hedgehog (SHH) protein delivery to prevent erectile dysfunction (ED) induced by cavernous nerve injury.^105^ SHH was proposed to regulate the increased collagen in penis of ED patients. Two strategies to introduce the SHH‐encapsulated PA hydrogels were investigated: (a) fast injection and subsequent gelation of PA solution containing SHH and CaCl_2_ (gelation stimuli) within the corpora cavernosa; (b) preformed PA hydrogels placed on top of an exposed cavernous nerve. In both cases, the SHH released from PA displayed a translational impact on suppressing the abnormal increase of collagen.

In summary, many peptide‐based supramolecular hydrogel systems have been explored as the carrier of therapeutic or characteristic proteins including growth factors, antibodies and other protein drugs. There were also many systematic studies conducted to understand the influence of physicochemical properties for both proteins and nanofibers on release kinetics. Most reports have focused on the local delivery of proteins by placing preformed peptide hydrogels at the desired site. The benefits of the self‐assembling peptide hydrogels as protein carriers can be summarized as follows: (a) large amounts of proteins can be easily incorporated into the peptide hydrogel networks under mild conditions; (b) the hydrogel networks can provide prolonged release for the encapsulated proteins; (c) the hydrogels can effectively shield the protein therapeutics within their three dimensional networks, thus maintaining their bioactivity until release and minimizing their potential toxicity; (d) the release profile can be well tuned by consideration of the protein size, the hydrogel mesh size, and the charge properties of both proteins and hydrogels; (e) in some cases, the hydrogels were speculated to increase the membrane permeability or enhance the therapeutic effect of the encapsulated proteins; (f) the peptide hydrogels can be designed to be injectable and shear‐thinning recoverable, and thus enable injection without syringe‐clogging or changes in properties of hydrogels. With these attractive and easily achieved advantages, self‐assembling peptide nanofiber hydrogels show great promise for future protein delivery applications.

## 8 Gene therapy

Successful gene therapy requires suitable vectors for local or systemic delivery of therapeutics to the desired sites. For example, the therapeutic efficiency of DNAs that encode for antigenic protein may be limited by rapid enzymatic degradation and fragmentation of DNA during the long circulation time and the poor cell permeability that results from the highly negatively charged phosphate backbone. Viral vehicles and non‐viral delivery systems such as gene gun, nanoparticles, and liposomes have been widely explored.^139^, ^140^, ^141^ However, these delivery systems may be accompanied by other disadvantages such as toxicity and impairment of DNA activity. A variety of peptide‐based systemic gene delivery particles which can complex DNA or RNA have been developed using functional peptide sequences such as cell‐penetrating peptides, endosome‐disrupting peptides, nuclear localization peptides, and so forth.^142^, ^143^, ^144^ However, these conjugation systems will inevitably introduce bifunctional cross‐linkers that may lead to toxicity and gene inactivation. For local delivery, peptide nanofibrous hydrogels with inherent biocompatibility show great potential to serve as the carriers with high loading for safe and efficient delivery of gene therapeutics.

**Figure 6:**
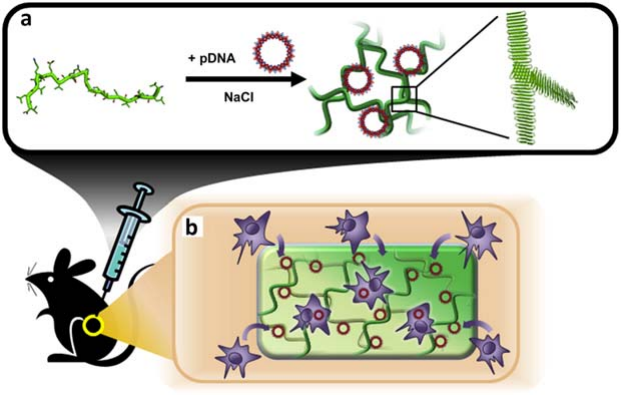
Schematic illustration of the DNA encapsulation into the *β*‐hairpin peptide hydrogels and the proposed delivery pathways following subcutaneous injection. (a) Incorporation of plasmid DNA into a *β*‐hairpin peptide supramolecular hydrogel. (b) Subcutaneous injection of DNA‐loaded hydrogels into the flanks of mice and subsequent infiltration of inflammatory cells. Used with permission from Ref. 145

Considering the high viscosity and low mobility of nanofiber networks, the local delivery of gene therapeutics through self‐assembling peptide hydrogels can largely facilitate the accumulation of genes in the tissue to which it is applied. Medina et al. studied the in vivo immunostimulatory potential of plasmid DNA encapsulated into β‐hairpin peptide hydrogels (Figure 6).^145^ Similar to proteins, the charges displayed on the DNA molecule can be properly utilized to control the process of encapsulation and release. When the peptides fold and self‐assemble into nanofibrillar networks triggered by increasing the ionic strength or temperature (to around 37°C), the anionic DNA in the solution could be efficiently entrapped within the networks via the electrostatic interactions with cationic side chains of lysine residues. Three peptide sequences MAX1 (+9), MAX8 (+7), and HTL2 (+5) with different net charges were shown to be able to encapsulate the HMGN1‐gp100 DNA vector and maintain its rheological properties during injection. It was demonstrated that a net charge of +5 is sufficient to retain a high amount of DNA and the more greatly charged peptide MAX1 may inhibit dissociation of the peptide and thus interfere with cellular internalization. An acute inflammatory response was caused by implantation of DNA‐loaded HLT2 gels into mice, leading to a large local concentration of cells that was demonstrated to facilitate DNA expression and antigen processing.

**Figure 7:**
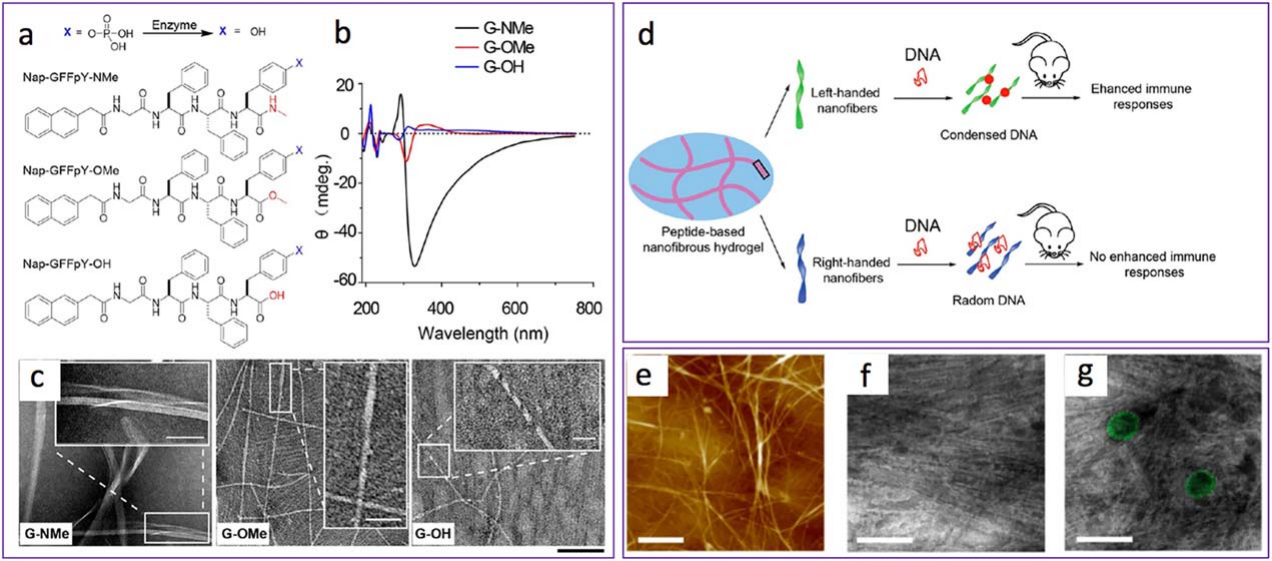
(a) Schematic illustration of enzymatic conversion and chemical structures of the precursors used to form peptide‐based nanofibrous hydrogels. (b) CD spectra of the three nanovectors. (c) TEM images of three nanovectors. G‐NMe shows right‐handed structure and G‐OMe and G‐OH show left‐handed structure corresponding to the difference in CD spectra. Scale bar: 100 nm (black); 50 nm (white, left); 25 nm (white, middle); 50 nm (white, right). (d) Process of β‐hairpin peptide hydrogels for enhancing immune responses of HIV DNA vaccines. AFM image (e) and TEM image (f) of Fmoc‐DDIKVAVK nanofibrous network. (g) TEM image of lentivirus (green) embedded within the branched arrangement of the individual fibers. Scale for AFM = 1 μm, TEM = 200 nm. (a)–(d) adapted with permission from Ref. 146, (e)–(g) adapted with permission from Ref. 153

Tian et al. reported the delivery of a DNA vaccine using a peptide‐based nanofibrous hydrogel to optimize the efficacy of HIV vaccine.^146^ The novel gelator (Nap‐GFFY‐NMe, G‐NMe) was designed and shown to undergo enzyme triggered gelation with an enhanced immune response. Compared with previously designed peptide gelators, Nap‐GFFY‐OMe (G‐OMe) and Nap‐GFFY‐OH (G‐OH) (Figures 7a–c), the *C*‐terminal methylamino modification of G‐NMe causes a structural transformation of the self‐assembled nanofibers from right‐handed structures to left‐handed, on which the DNA could be effectively condensed (Figure 7d). As stated by the author, this hydrogel nanovector‐induced DNA condensation could promote the DNA transfection and thus strongly activate both humoral and cellular immune responses against HIV by intramuscular, intradermal, or subcutaneous injection. Their results suggest that the peptide‐based hydrogel nanovector has great potential for the delivery of HIV DNA vaccines and could be applied to other immunotherapies.

Extensive efforts have been focused on virus vectors such as murine leukemia virus for clinical gene therapy.^147^, ^148^, ^149^ However, unwanted issues such as pathogenicity, immune response, and inflammatory reactions need to be addressed. Ideal viral vectors require capabilities of low genotoxicity and immunogenicity as well as highly efficient delivery.^147^ Recent advances include adeno‐associated virus and lentivirus with reduced genotoxicity and enhanced gene delivery.^150^, ^151^, ^152^ From another perspective, the genotoxicity of viral vectors could be avoided by developing a second carrier for the protection of viral vectors that can constrain its mobility. In a recent work, self‐assembling Fmoc‐peptide hydrogels (Fmoc‐DDIKVAVK) were designed as viral vector gene delivery vehicles for localized gene therapy (Figures 7e–g).^153^ The peptide sequence was carefully selected to non‐covalently interact with the viral membrane and contain viral activity to the site of injection. mCherry lentivirus‐loaded self‐assembling Fmoc‐peptide hydrogels that were injected into the mouse brain showed the least number and volume of mCherry+ cells in the host striatum delivery of virus alone. However, the similar density of mCherry+ cells proved that the transduction efficiency of the delivered virus was remained after releasing from the hydrogel networks. This novel method that combines the advantages of viral vectors and peptide hydrogels will enable the localized and efficient delivery of gene therapeutics. Although little progress has been made to use self‐assembling peptide hydrogels for gene delivery, the pioneering works described above demonstrate their promising potential as non‐viral gene vectors to delivery more types of gene therapeutics and promote gene therapies.

## 9 Future perspective

In this review, we discussed the design principles of peptide‐based supramolecular hydrogels in the context of protein delivery and gene therapy. The great diversity of amino acid sequences provides the possibility to fine‐tune supramolecular interactions to form hydrogels of various properties. These hydrogels possess a great potential as effective biologics carriers due to their inherent biocompatibility and tunable biodegradability. Compared to the extensive studies focused on the protein delivery, little was done on the use of self‐assembling peptide hydrogels for gene delivery. For future development of peptide‐based hydrogels for clinical translation, there are a few noteworthy points. First, the toxicity and biocompatibility of peptide‐based hydrogels should be evaluated in a comprehensive manner since the biocompatibility is the basic requirement for in vivo applications.^154^ It is well known that assembled peptides are much more resistant to enzymatic degradation than the monomeric ones.^155^ There might also be a difference in toxicology between peptides in the assembled form and peptides in the soluble form. Second, an in‐depth understanding of the biologics' release profile is essential for further optimization of the therapeutic outcomes. This would need rational and quantitative correlations of the physicochemical properties of the biologics (surface charge, water dispersity) with the network structures (mesh size, filament alignment, etc.) and materials properties (degradation rates, stiffness, surface chemistry, etc.). More efforts should also be devoted to developing hydrogels systems capable of stimuli‐specific release of biologics as well as enzyme‐specific degradation of the hydrogels.^155^, ^156^ Third, it is important to incorporate biologically active peptides into the hydrogel design to explore synergistic combination of peptide drugs/epitopes with biologics. Such bioactive peptides could be used to identify a particular type of cells such as cancer cells, to co‐stimulate cells for proliferation, migration, or differentiation, or simply to allow for cell adhesion.

Lastly, self‐assembling peptide hydrogels have been mostly used in local delivery due to their high viscosity and low mobility. Although local delivery is able to attain higher concentrations of the biologics at the desired sites without necessarily involving a targeting strategy, systemic delivery is sometimes the more preferred approach due to its ease of administration, low invasive property, and better patient compliance. For this purpose, more effort should be devoted to investigating the peptide‐based nanogel system for systemic delivery, as inspired by the recently developed polymeric nanogels. The nanogels are nanosized crosslinked networks mostly composed of synthetic polymers and are very promising as biologics carriers because of their high loading capacity, high stability, and responsiveness to environmental factors.^157^, ^158^, ^159^, ^160^ They are mostly produced by polymerization of monomers in nanoscale heterogeneous environments stabilized by surfactants or cross‐linking of preformed polymers. These polymer‐based nanogels have been largely investigated for protein and gene delivery.^161^, ^162^, ^163^ The self‐assembling peptide nanogels may be formed by self‐assembly and gelation in a confined heterogeneous nano‐environment and are expected to maintain their advantageous properties in systemic delivery of biologics. Further noteworthy studies include the development of self‐assembling peptide nanogels and incorporation of targeting properties by adding specific peptide sequence for targeting or bioresponsive release. Although a long way ahead, we believe that self‐assembling peptide hydrogels will exert significant influence on the efficient delivery of biologics.
